# Efficacy of psychotherapy versus pharmacotherapy, or their combination, in chronic depression: study protocol for a systematic review and network meta-analysis using aggregated and individual patient data

**DOI:** 10.1136/bmjopen-2024-089356

**Published:** 2025-02-19

**Authors:** Elisabeth Schramm, Moritz Elsaesser, Julia Müller, Nana-Adjoa Kwarteng, Theodoros Evrenoglou, Pim Cuijpers, Efthimiou Orestis, Daniel N Klein, Martin B Keller, Toshi A Furukawa, Adriani Nikolakopoulou

**Affiliations:** 1 Department of Psychiatry and Psychotherapy, Medical Center – University of Freiburg, Faculty of Medicine, University of Freiburg, Freiburg, Germany; 2 Institute of Medical Biometry and Statistics, Faculty of Medicine and Medical Center, University of Freiburg, Freiburg im Breisgau, Germany; 3 Department of Clinical, Neuro and Developmental Psychology, WHO Collaborating Centre for Research and Dissemination of Psychological Interventions, Vrije Universiteit Amsterdam, Amsterdam, The Netherlands; 4 International Institute for Psychotherapy, Babeș-Bolyai University, Cluj-Napoca, Romania; 5 Institute of Primary Health Care (BIHAM), University of Bern, Bern, Switzerland; 6 Department of Psychology, Stony Brook University, Stony Brook, New York, USA; 7 Department of Psychiatry and Human Behavior, The Warren Alpert Medical School of Brown University, Providence, Rhode Island, USA; 8 Department of Health Promotion and Human Behavior, Kyoto University Graduate School of Medicine/School of Public Health, Kyoto, Japan

**Keywords:** Systematic Review, Meta-Analysis, Network Meta-Analysis, Depression & mood disorders, Adult psychiatry, PSYCHIATRY

## Abstract

**Abstract:**

**Introduction:**

Chronic depression represents a common and highly disabling disorder. Several randomised controlled trials (RCTs) investigated the effectiveness of psychological, pharmacological and combined treatments for chronic depression. This is the first overarching systematic review and network meta-analysis (NMA) based on aggregated and individual patient data comparing the efficacy and acceptability of various treatment options for all subtypes of chronic depression. Furthermore, individual demographic and clinical characteristics that predict or moderate therapy outcomes will be investigated.

**Methods and analysis:**

A systematic literature search of the Cochrane Library, MEDLINE via Ovid, PsycINFO, Web of Science and metapsy databases will be conducted from database inception without language restrictions to include all available samples from RCTs that investigated the efficacy of psychotherapy versus pharmacotherapy, or their combination in adult inpatients or outpatients with a primary diagnosis of chronic depression. Exclusively internet-based treatment studies will be excluded. The main outcome is depression severity measured on a continuous observer-rated scale for depression at 6 months post-treatment (range 3–12 months). Two reviewers will independently screen and select eligible studies based on the predefined inclusion and exclusion criteria. Risk of bias will be assessed using version 2 of the Cochrane risk-of-bias tool for randomised trials (RoB 2). Individual patient data (IPD) will be requested and incorporated in the network when provided, as it is the gold standard of evidence. For studies which do not provide IPD, aggregate data (AD) will be extracted and incorporated in lieu of IPD for the NMA, strengthening the evidence base and leveraging all existing evidence regardless of data availability restrictions. An NMA comparing psychotherapies and a network meta-regression estimating individualised treatment effects of psychotherapy will be implemented assuming a Bayesian framework. All models will be fitted in R with calls to JAGS. Empirical informative prior distributions will be used for model parameters where available, and non-informative priors will be used in cases where empirical priors are not available.

**Ethics and dissemination:**

This IPD-NMA requires no ethical approval. All results will be disseminated as peer-reviewed publication in a leading journal in this field and presented at (inter)national scientific conferences.

**PROSPERO registration number:**

CRD42024526755.

**STRENGTHS AND LIMITATIONS OF THIS STUDY**

- To include all available randomised controlled trials and maximise statistical power, we will perform a network meta-analysis (NMA) and synthesise evidence based on both aggregated data (AD) and individual participant data (IPD).
- The results will offer guidance about the most effective treatment approaches for clinicians and patients seeking optimal individualised management strategies.
- A network meta-regression model adjusted in terms of individual demographic and clinical characteristics, that impact therapy outcomes, will be fitted to yield individualised treatment recommendations.
- The IPD-NMA will not be able to examine variables that have not been measured in the original studies.
- The evidence base may yield different forms of sparse data such as sparse networks, small number of studies and small subgroups, which will likely lead to persisting uncertainty around synthesised estimates of primary and secondary outcomes.

## Introduction

 Chronic depression is a common and long-term disabling disorder.^1^ Up to one-third of all depressive disorders take a chronic course^2^ with the definition of chronic varying in the literature regarding duration (between a minimum of 1–3 years), severity (dysthymia, chronic major depression) and the type of course since the first onset (dysthymia with or without superimposed major depressive episode(s), chronic major depression or recurrent major depression without interepisode recovery).^3^ In 2013, the Diagnostic and Statistical Manual of Mental Disorders, Fifth Edition (DSM-5) merged these various presentations into the rubric of persistent depressive disorders (PDD) with defined diagnostic criteria present for at least 2 years and four coding specifiers. Unlike major depressive disorder, chronic depression tends to have a more subtle but persistent presentation, leading to comparably more significant impairments in functioning and more reduced quality of life.^4^ Despite new developments of treatment approaches in the past decades, chronic depression remains challenging to treat, with varying degrees of success reported for different modalities. Common treatment complicating factors such as comorbidity with mental and physical disorders, poor social integration, early onset or a history of early trauma lead to a high rate of treatment-resistance.^5^

Psychotherapy and pharmacotherapy are the two primary treatment modalities used to manage chronic depression. Psychotherapy encompasses various approaches such as the Cognitive Behavioural Analysis System of Psychotherapy (CBASP)^6^,^8^ as the only model specifically designed for chronic depression, and more general methods such as Cognitive-Behavioural Therapy (CBT)^9^, Interpersonal Therapy (IPT)^10^ or Psychodynamic and Psychoanalytic Therapy.^11 12^ Pharmacotherapy, as the most commonly used treatment, involves antidepressant medications including selective serotonin reuptake inhibitors, serotonin-norepinephrine reuptake inhibitors, tricyclic antidepressants, and others, to modulate neurotransmitter activity in the brain.

Furthermore, psychotherapy and pharmacotherapy are integrated into combination therapy to potentially yield faster and better treatment outcomes. Despite the availability of these treatment options, uncertainty remains regarding their relative efficacy and differential response in managing chronic and treatment-resistant depression. While some studies suggest that combination therapy may be more effective than either psychotherapy or pharmacotherapy alone,^13 14^ others indicate that certain monotherapies are particularly suitable for specific patient profiles or severity levels.^15^ The last comprehensive meta-analysis specifically about chronic depression dates from 2014 and recommends different approaches depending on the subtype of chronic depression.^12^ However, within the last 10 years, several new studies have appeared that have not yet been synthesised with the previous evidence (eg, Refs).^16^,^22^ An individual participant data network meta-regression (NMR) investigated the efficacy of CBASP, pharmacotherapy or their combination and several effect moderators, but included only three studies.^15 23^ In a fairly recent network meta-analysis (NMA) on adult depression, a subgroup analysis showed superior effects for combined treatment versus psychotherapy or pharmacotherapy.^14^ However, this subgroup analysis did not distinguish between chronic courses and treatment-resistant depression and did not consider long-term effects.

Therefore, an up-to-date comprehensive systematic review on the efficacy of psychotherapy versus pharmacotherapy, and their combination in treating chronic depression is urgently needed. Our NMA will incorporate all available randomised controlled trials (RCTs) to capture the full breadth of evidence.^24^ By analysing individual patient data (IPD) as well as aggregate data (AD; if IPD is not available) from a range of studies, we aim to obtain insights into the most effective treatment approaches, identify potential predicting and moderating factors, and offer guidance for clinicians and patients seeking optimal management strategies. The results of this meta-analysis may have significant implications for the individualised treatment of chronic depression, informing clinical practice and shaping future research in this field.

### Objectives

The aim of this systematic review is to evaluate the efficacy of psychological versus pharmacological, or combination treatments in chronic depression. To this end, the proposed systematic review will answer the following questions:

1. Which psychological versus pharmacological, or combination treatments are most effective for chronic depression?
2. How do individual demographic and clinical characteristics predict or moderate individualised treatment recommendations?

## Methods and analysis

### Eligibility criteria

We will include data from RCTs that investigated treatment effects in adult (>18 years old) inpatients or outpatients with a primary diagnosis of chronic depression. Depressive courses are defined as chronic when lasting for at least 2 years (according to the definition of the classification systems of DSM-5 and ICD-11) including PDD (DSM-5), chronic major depression, double depression (dysthymia with superimposed major depressive episode), recurrent major depression with incomplete interepisode recovery, dysthymia or any corresponding conditions according to standard operationalised diagnostic criteria as the primary diagnosis. Comorbid disorders are allowed and all respective information will be collected.

The treatments of interest in our IPD-NMA include the following:

- Different types of psychotherapies (including CBASP, Cognitive-Behavioural Therapy, Interpersonal Therapy, Modular-Based Psychotherapy, Metacognitive Training, Dialectical Behaviour Therapy, Mindfulness-based Cognitive therapy (Long-term) Psychoanalytic Psychotherapy (Brief) Supportive Psychotherapy, Group Person-based Cognitive Therapy, Cognitive Therapy, Problem-solving Treatment and Schema Therapy).
- Antidepressant pharmacotherapy as a comparator to psychotherapy including any of the antidepressive agents licensed for the treatment of major depression in the country where the trial was conducted.
- Different types of psychotherapies mentioned above as an adjunct treatment to other treatments, for example, treatment as usual (TAU), care as usual (CAU), exercise, counselling.
- Different types of psychotherapies mentioned above as an adjunct treatment to any type of antidepressant pharmacotherapy (combination treatment).
- If waitlist control is included in the screened studies, it will be used as the primary reference category when reporting relative treatment effects. CAU and TAU conditions will be compared with each other and examined with regard to their similarity. If substantial differences between TAU and CAU are detected, we will consider whether they need to be split into meaningful categories. Otherwise, they will be merged and treated as one single treatment (ie, TAU/CAU) in the statistical analyses.
- Only studies implementing face-to-face psychotherapy will be included; exclusively internet-based treatment studies will be excluded.

### Information sources and search strategy

We will first conduct an electronic literature search of the Cochrane Library, MEDLINE via Ovid, PsycINFO, Web of Science and metapsy databases with the keywords ‘psychotherapy’, ‘chronic depression’ and all related terms. Initial searching will be conducted in March 2024. Searches will be rerun just before the final analyses and any further studies identified will be retrieved for inclusion. There will be no restriction for the publication period. As an example, this is the final search string for OVID:

((chroni*5 adj3 depress*).ti,ab. OR (chroni*5 adj3 MDD).ti,ab. OR exp Depressive Disorder, Treatment-Resistant/OR exp Dysthymic Disorder/OR (treatment-resistan* adj3 depress*).ti,ab. OR (treatment-resistan* adj3 MDD).ti,ab. OR (therapy-resistan* adj3 depress*).ti,ab. OR (therapy-resistan* adj3 MDD).ti,ab. OR (dysthymia or (dysthym* adj2 disorder*1)).ti,ab. OR (persist* adj2 depress*).ti,ab. OR (persist* adj2 MDD).ti,ab. OR (double depression).ti,ab.) AND (exp Psychotherapy/or psychotherap*.ti,ab. OR (psychologi* adj3 treatmen*2).ti,ab. OR (psychologi* adj3 interventio*2).ti,ab. OR CBASP.ti,ab. OR ((cognitive adj2 therapy).ti,ab. OR (behavior* adj1 therapy).ti,ab. OR mindfulness.ti,ab. OR psychoanalytic.ti,ab. OR psychodynamic.ti,ab. OR (schema therapy).ti,ab.)

In accordance with the Cochrane handbook, preprints will be considered a potentially relevant source of study evidence and will therefore be assessed for eligibility. Backward and forward citation searches of included studies and relevant reviews will be performed. To find references in the grey literature, we will contact authors of included studies and relevant conference abstracts of unpublished studies. Furthermore, we will use relevant mailing lists, for example from the Society for Psychotherapy Research or (inter)national professional associations, to draw attention to this project.

### Study selection

At least two reviewers will independently screen the title and abstract of all records of the systematic literature search and select eligible studies based on the predefined inclusion and exclusion criteria. If both reviewers independently determine that a study may be eligible based on title and abstract screening, then a full-text article review will be completed. Disagreements between individual judgements will be resolved via discussion with a third reviewer. In case of ongoing disagreement, a meeting will be held with the complete study team involving all reviewers allowing them to present the reasoning for their judgement. Consensus should be reached after debate and decisions will be documented in written form.

### Data extraction

The IPD of the originally established data sets will be requested from the authors of all eligible studies. One person will check the received data for completion. All obtained IPD will be cleaned, coded and saved in appropriate files to make the data as uniform as possible. Afterwards, we will compare the published data of each data set (ie, numbers and percentages, or means and SD of baseline demographics as well as clinical variables) with the summaries that we will obtain directly from the IPD. Any major inconsistencies will be discussed and followed-up. Corrections will be made as necessary. In cases where IPD are not provided within a reasonable time after the request, AD will be extracted from the publication and used instead. Two individuals will be responsible for performing the data extraction. One extractor will perform the data extraction, while the second will perform the quality control and ensure that all data are properly extracted.

When extracting AD, the following data points will be collected:

- Study-level data: year of publication; validated depression scale(s) used in study.
- Participants: mean age at baseline; mean age at first depressive episode; mean duration of chronic depression; proportion of participants with prior antidepressant treatment; proportion of participants with current antidepressant treatment; proportion of participants with prior psychotherapeutic treatment; other covariates (eg, childhood maltreatment, loneliness, attachment style, personality, rejection sensitivity, mentalisation, emotion regulation, comorbidities, severity, interaction style, avoidance, social functioning, alexithymia empathy).
- Outcomes per arm: mean follow-up duration; mean baseline, post-treatment and follow-up depression score, and SD; mean change from baseline depression score and SD; number of participants at baseline, post-treatment and follow-up; percentage of dropouts (treatment discontinuation); side effects; and (serious) adverse events.

In case of repeated measures, the time point of each repeated measure will also be extracted. Disagreements between individual judgements will be resolved via discussion. In case of discrepancies, a meeting will be held involving both extractors allowing them to present the reasoning for their judgement. A consensus should be reached after debate and decisions should be documented in meeting minutes. Data will be recorded in excel spreadsheets.

### Outcomes

The main outcome is depression severity measured on a continuous observer-rated scale for depression at 6 months post-treatment (range 3–12 months). If the respective study reports results at two or more time points within this frame, we will prioritise the time point closest to 6 months. If time points are equidistant, we will use the later one. Where different scales such as the Montgomery-Asberg Depression Rating Scale (MADRS)^25^, the Quick Inventory of Depressive Symptomatology- Clinician Rating (QIDS-C)^26^ or different versions of the Hamilton Rating Scale for Depression (HAM-D/HRSD)^27^ are reported, we will attempt to transform their respective scores to the 17-item HRSD score^28 29^ as the most frequently utilised measure^30^ using conversion procedures (eg, http://ids-qids.org/interpretation.html or)^31^.

Secondary outcomes are:

1. Treatment response, defined as 50% or greater reduction from baseline to study endpoint in the study’s primary observer-rated or self-rated depression scale.
2. Remission, defined as scoring below the validated thresholds of the study’s primary observer-rated or self-rated depression scale at endpoint.
3. Study drop-out for any reason, as a proxy measure of overall treatment acceptability.
4. Study drop-out due to side effects.
5. Depression severity as measured on a continuous self-rating scale for depression, such as Beck Depression Inventory-II (BDI-II)^32^ or Inventory of Depressive Symptomatology, Self-Report (IDS-SR)^33^. Different scales will be converted into BDI-II using conversion tables and equipercentile linking.^34^
6. Global Assessment of Functioning (GAF).
7. Social functioning, as measured by any validated measure for impaired social functioning such as the Social Adjustment Scale-Self-Report (SAS-SR)^35^.
8. Quality of Life, as measured by any validated measure for life quality such as the WHO Quality of Life (WHOQOL^36^) or the Short Form 36 Health Survey (SF-36).^37 38^
9. Side effects.
10. Adverse and serious adverse events.

### Risk of bias assessment

We will assess risk of bias (RoB) in the included studies using the Cochrane RoB 2 tool for randomised trials.^39 40^ The assessment will be done by two independent raters who have successfully completed an official RoB 2 workshop by Cochrane Germany. If raters disagree, the final rating will be made by consensus with involvement of another member of the review group (if necessary). We will evaluate RoB in the following domains: bias arising from the randomisation process, bias due to deviations from intended interventions, bias due to missing outcome data, bias in measurement of the outcome and bias in selection of the reported result.

Please note that, when IPD are available, the RoB assessments can be different from those based on AD. For example, even when the original authors used alternative imputation methods for handling missing outcomes (eg, last observation carried forward), we can apply different imputation methods more suitable for the current use case (eg, multiple imputation) on the IPD. We will assess the RoB for the outcomes used for the primary outcome of our NMA.

### Data synthesis

Measurements of depression severity are often reported on validated continuous observer-rated scales for depression. If all selected studies report depression severity using scales with defined conversion factors, we will attempt to transform them into the 17-item HAM-D and use the mean difference (MD) between treatment groups in the specific study as the effect measure for the primary outcomes. In studies where multiple scales are reported, all scales reported in the articles will be extracted and those that can be converted to HAM-D will be used for the analysis. In cases where multiple scales are reported and more than one can be converted to HAM-D, or if neither scale can be converted, the scale that is more commonly used across studies will be selected for consistency and comparability. However, if depression severity is measured via an alternative scale, we will standardise all the study-specific means and synthesise the data by using the standardised mean difference (SMD) as our effect measure. For binary outcomes, odds ratios (ORs) will be used as effect measure. In such cases, studies reporting zero events in all treatment arms will be excluded from the analysis. Studies that provide IPD, but do not report outcomes for more than 50% of the participants, will be excluded from the analysis of the relevant outcome. In studies providing IPD with a missing outcome for less than or equal to 50% of the participants, we will use multilevel models to borrow information across studies and perform multiple imputations at the network level, which involves borrowing information across studies while allowing for heterogeneity and clustering in a multilevel imputation model.^41^ AD reporting studies that do not report a given outcome will be excluded from the analysis of the relevant outcome.

#### Transitivity checks

The validity of our NMA relies on the validity of its core assumption, namely the assumption of transitivity. This requires that all the characteristics of the included studies that act as effect modifiers have a balanced distribution across all treatment comparisons. To perform such assessments, we plan to examine the distribution of the extracted effect modifiers using boxplots (for continuous characteristics) and barplots (for categorical characteristics)

#### Network meta-analysis

To capture the clinical and methodological differences that inevitably occur among the included studies, we will fit random-effects models. For simplicity we will assume a common heterogeneity parameter across all the available treatment comparisons.

If IPD is provided for all studies, IPD-NMAs will be conducted for the overall treatment effects and the overall treatment adherence. All analyses will be conducted in R with calls to JAGS via the rjags package for the implementation of Bayesian hierarchical models using vague priors for all location parameters (effect sizes and regression coefficients).

The IPD-NMA will be implemented based on the model described by Saramago *et al* (model 2), and adapted as needed depending on whether the outcome is continuous or binary.^42^ We will assume the following model for the case of a two-arm study:

$$y_{ijk}=\left\{ \begin{array}{l} \mu_{jb},if\;k=b\\ \mu_{jb}+\delta_{jbk},if\;k\neq b \end{array}\right.$$

$$\delta_{jbk}∼N\left(d_{bk},\tau^{2}\right)$$

(1) $$d_{kq}=d_{kb}-d_{qb}$$

Where $y_{ijk}$ denotes the observed outcome measure, i, j and k are the patient, study and treatment indices, respectively. b denotes an arbitrarily chosen reference treatment. q represents a treatment in the network which is neither k or b. $\mu_{jb}$ is the mean outcome in the reference group in study j, and $\delta_{jbk}$ represents the average relative treatment effect between treatment b and treatment k. $\delta_{jbk}$ is assumed to be normally distributed across studies around a mean of $d_{bk}$, with a variance of random effects $\tau^{2}$, assumed to be common for all comparisons. For binary outcomes, $y_{ijk}$ will be assumed to come from a Bernoulli distribution and $\delta_{jbk}$ will correspond to the log OR of treatment k and treatment b. For multiarm trials, the equation above will need to be extended to multivariate normal distributions.

If IPD is not available for one or more of the included studies, we will use the respective published AD. Available IPD will be synthesised together with AD from studies for which IPD is not available. The IPD/AD-NMA will be implemented in a three-step Bayesian hierarchical model in R, with calls to JAGS for the implementation of Bayesian hierarchical models to estimate the overall treatment effects and the overall treatment adherence. Models will use informative priors for heterogeneity when available. If informative priors are not available for heterogeneity and for all other location parameters, vague priors will be used. The model, based on Saramago *et al* (model 3) is similar to equation 1.^42^ However, in the IPD/AD-NMA for binary outcomes, $y_{ijk}$is assumed to come from a binomial distribution for AD or a Bernoulli distribution for IPD. $d_{bk}$ is determined using both the AD and IPD and the associated heterogeneity considers the heterogeneity across both sources of data.

Finally, across treatments effect estimates and their SEs will be considered in order to calculate the final relative ranking of the different competing treatments. To do so, we will rely on the ranking metric defined according to the Surface Under the Cumulative Ranking curve (SUCRA)of each treatment.^43^

#### Consistency checks and heterogeneity estimation

To assess the statistical manifestation of transitivity, namely the consistency assumption, we plan to employ both local and global checks for consistency. For local checks, we will implement the Separating Indirect from Direct Evidence (SIDE)approach to evaluate the difference between direct and indirect estimates for each treatment comparison in the network that provides both of this sources of information, Global checks will be implemented using the design-by-treatment interaction model.

#### Network meta-regression (NMR)

The literature suggests many candidates for prognostic factors (variables associated with response regardless of the treatment) and for effect modifiers (variables associated with differential response depending on the treatment) in the treatment of depression. However, we will only include variables in our analyses if they are particularly pertinent in the differential treatment of chronic depression in the context of psychological and pharmacological treatments. The variables will first be limited by their availability in the included original studies. When several variables that measure similar aspects are available, the research team will discuss and reach consensus on the most important predictors and decide on which should be included in the model. For systematically missing covariates, missing data will be described and reasons for missing data will be explored. If 30% of a variable’s data is missing, we will consider imputation. If such a scenario arises, we will assess the appropriateness of multiple imputation methods and explore the impact of missing data on conclusions about the comparative effects on the primary outcome in sensitivity analyses. We will fit a penalised regression model, for example, Bayesian LASSO or ridge regression. As in the NMA, models will use empirical priors for heterogeneity when available and non-informative priors (eg, $N({0,100}^{2})$) for all location parameters otherwise. The NMR model will assume independent, unrelated treatment–covariate interactions for each treatment comparison between treatment effects and covariates.

If IPD is provided for all included studies, the IPD-NMR will be implemented based on the model described by Jansen *et al* (model 1).^44^ We will use the following model, in which only one covariate is shown for simplicity:

$$y_{ijk}=\left\{ \begin{array}{l} \mu_{jb}+\beta_{0j}x_{ij},if\;k=b\\ \mu_{jb}+\delta_{jbk}+\beta_{0j}x_{ij}+\beta_{1bk}x_{ij},if\;k\neq b \end{array}\right.$$

$$\delta_{jbk}∼N\left(d_{bk},\tau^{2}\right)$$

$$d_{kq}=d_{kb}-d_{qb}$$

(2) $$\beta_{1,kq}=\beta_{1,kb}-\beta_{1,qb}$$

Where i, j, k,q and b are as defined for equation 1. $x_{ij}$ is the value of the covariate for individual i in study j, $\beta_{0j}$ is the study-specific estimated prognostic effect of x and $\beta_{1bk}$ is the interaction between $x_{ij}$ and the relative treatment effect b versus k. In this model, $\mu_{jb}$ denotes the mean outcome for the control group of the study when the covariate value is 0, and $\delta_{jbk}$ denotes the effect size between treatment b and treatment k in a study when the covariate value is 0. Binary outcomes and multiarm studies will be handled as in the case of IPD-NMA.

If IPD is not available for a subset of the included studies, we will use the respective published AD and synthesise them together with IPD providing studies. The IPD/AD-NMR will be implemented based on the model described by Jansen *et al* (model 2)^44^ as follows:

IPD

$$y_{ijk}=\left\{ \begin{array}{l} \mu_{jb}+\beta_{0j}x_{ij},\;if\;k=b\\ \mu_{jb}+\delta_{jbk}+\beta_{0j}x_{ij}+\beta_{1bk}x_{ij},\;if\;k\neq b \end{array}\right.$$

AD

$$y_{ijk}=\left\{ \begin{array}{l} \mu_{jb},\;if\;k=b\\ \mu_{jb}+\delta_{jbk}+\beta_{1bk}x_{j},\;if\;k\neq b \end{array}\right.$$

$$\delta_{jbk}∼N\left(d_{bk},\tau^{2}\right)$$

$$d_{kq}=d_{kb}-d_{qb}$$

(3) $$\beta_{1,kq}=\beta_{1,kb}-\beta_{1,qb}$$

Where i, j, k, q, and b are as defined for equation 1. For the IPD portion, $x_{ij}$, $\beta_{0j}$, $\beta_{1bk}$, $\mu_{jb}$, $\delta_{jbk}$ and $y_{ijk}$ are as defined in equation 2. For the AD subset, $x_{j}$ is the average of the covariate in study j and $\beta_{1bk}$ is the interaction of $x_{j}$ for treatment b relative to treatment k for the aggregated covariate. The definition for $\mu_{jb}$ is the effect size for the control group of the study, and $\delta_{jbk}$ represents the effect size between treatment b and treatment k in a study when the covariate value is 0. For binary outcomes,$y_{ijk}$ and $\delta_{jbk}$ will be handled as in the case of IPD/AD-NMA. To enable estimation using both IPD and AD, this model assumes that the treatment-by-covariate interaction regression coefficients, $\beta_{1bk}$, are the same for individual-level covariates from IPD and study-level covariates for AD.

We will use the estimated parameters of the IPD-NMA or IPD/AD-NMA model to create a prediction model in order to provide personalised predictions according to the covariates considered. The model will take patient-specific values for covariates as inputs and provide a prediction of the outcome under each treatment of interest. Initial nodes will be defined as stated in the eligibility criteria of the protocol. If not enough data exist for making predictions for psychotherapy types, we will merge nodes to

- any type of psychotherapy,
- antidepressant pharmacotherapy,
- any type of psychotherapies as an adjunct (TAU, CAU, exercise, counselling, electroconvulsive therapy),
- any type of psychotherapy as an adjunct treatment to any type of antidepressant pharmacotherapy,
- waiting list, CAU, TAU either alone or as an adjunct to any type of antidepressant pharmacotherapy

To assess the predictive performance of this model, we will use an internal–external cross-validation method. This involves taking one study out at a time, developing the model using the remaining studies and testing the model on the left-out study. Then, we will assess the model performance in terms of calibration and discrimination for benefit, and decision accuracy, using recently developed methods.^45^,^47^

Subgroup analyses will be performed for study-level characteristics defined above. Study-level characteristics will be explored using meta-regression in a Bayesian framework with informative priors where available or vague priors in all other cases. If subgroup analyses cannot be undertaken due to small number of studies, we will conduct separate pairwise meta-analyses for the small subgroups and provide descriptive statistics to offer insight regarding the subgroup. In the particular case where subgroup analyses result in disconnected subnetworks, that is, there are pairs of treatments that neither direct nor indirect estimates can be derived, we will employ a component NMA (cNMA) in an attempt to connect the disconnected networks consisting the small subgroup and facilitate analysis.

### Meta-biases

We will examine the existence of possible small-study effects and publication bias both in terms of the pairwise and network level. Pairwise examinations will be held by using the contour-enhanced funnel plots for each pairwise comparison with at least 10 studies available, while for examinations at the network level, we will use the comparison-adjusted funnel plot and plot all the study-specific treatment effects in a unique graph. In order to evaluate potential availability bias with regard to the available participant-level covariates included in the model, covariate effects will be estimated in IPD studies and compared with AD studies. If considerable differences are found, this will be discussed in the final publication.

### Confidence in meta-analytical estimates

To rate the quality of the best available evidence and provide a comprehensive summary, the Grading of Recommendations, Assessment, Development and Evaluation (GRADE) approach will be used. The quality of evidence will be assessed across the domains of RoB, consistency, directness, precision and publication bias. Additional domains may be considered where appropriate.

When evaluating the confidence in the NMA for the primary outcomes, we will use the six domain CINeMA framework,^48^ which considers within-study bias, across-studies bias, indirectness, imprecision, heterogeneity and incoherence when evaluating the confidence in the NMA. Since there is no current consensus regarding the definition of clinically important effect size for change in depression severity in chronic depression, the clinically important effect size for clinical equivalence will be based on statistical difference.

### Patient and public involvement

None.

## Ethics and dissemination

As a systematic review of published studies, this IPD-NMA requires no ethical approval. In the event that changes to the protocol occur, changes will be reported in protocol amendments in PROSPERO. All results will be disseminated as peer-reviewed publication in a leading journal in this field and presented at (inter)national scientific conferences.

## Discussion

This protocol describes a systematic review and NMA on the efficacy of psychotherapy versus pharmacotherapy or their combination in the treatment of chronic depression. As the most recent comparable systematic reviews on chronic depression are several years old, do not combine IPD and AD, and do not include various important studies,^12 15^ an up-to-date overview is urgently needed. To include all available RCTs and maximise statistical power, our NMA approach will allow direct and indirect comparisons based on AD as well as IPD.

In some cases, IPD meta-analysis has demonstrated the potential to produce better quality, more precise and more reliable results than meta-analysis of only AD.^49^,^52^ However, it is often difficult to access IPD, and subgroup analyses of interest within IPD often will be small.^42 44 53^ In order to maximise the use of the available information and attempt to reduce the overall uncertainty, complex methods that allow the inclusion of both IPD and AD can be used to evaluate overall treatment effects, irrespective of subgroups of interest. Then, regression techniques within the combined IPD and AD can enable the examination of the association between treatment effects and potential covariates.

Despite the planned combination of AD and IPD, the expected number of applicable studies fulfilling the defined inclusion and exclusion criteria is small. Thus, any subgroup analysis performed from the identified studies is also expected to be limited by small numbers and therefore to be low powered. The updated NMA will improve our understanding of the therapeutic landscape. However, uncertainty in the estimation of outcomes will likely persist.

It is important to note that, despite attempts to access the IPD of all identified studies, the possibility remains that some studies will not be able to provide the IPD. This could lead to an availability bias with regard to the available participant-level covariates included in the model. Furthermore, since the NMA is based on existing studies, the analysis can only examine potential predictors or effect modifiers that are measured in the original studies. For AD, this data availability is further limited to the variables that are reported in the original publication. Therefore, it is currently unclear whether all variables of interest defined in the protocol will be incorporated in the NMR model. The use of MDs would enhance the clinical interpretability of the meta-analysis results if all scales used in the original studies are convertible to the HAM-D. However, if alternative scales are used, it will be necessary to use SMDs to obtain estimates of treatment efficacy, which will limit the clinical interpretability of the results.

This systematic review and meta-analysis will focus on the inclusion of RCTs, since they are considered less susceptible to known and unknown confounders due to the randomisation and have well defined criteria, treatments and endpoints. We plan to complete the systematic literature search, obtain aggregated or individual participant data from the relevant studies and conduct the statistical analyses by mid-2025. We will aim for the results to be submitted to an international peer-reviewed journal by the end of 2025. The code behind our results will be made accessible through a public GitHub repository. These codes could then potentially be used to create an interactive tool (eg, an R-Shiny application) that will allow using our prediction model and visualise its results without requiring any programming expertise for users. Ultimately, this systematic review and meta-analysis aims to help elucidate and estimate the impact of treatment alternatives, and associated predictive and effect modifying factors, on chronic depression to enable customised treatment strategies for patients experiencing chronic depression.
