# Supplementary material for: Efficacy of psychotherapy versus pharmacotherapy, or their combination, in chronic depression: study protocol for a systematic review and network meta-analysis using aggregated and individual patient data
Source: BMJ Open. 2025 Feb 19;15(2):e089356. doi: 10.1136/bmjopen-2024-089356 (PMC11840898; doi:10.1136/bmjopen-2024-089356)
Supplement: Supplementary file 1 [file bmjopen-2024-089356.reviewer_comments.pdf]

PEER REVIEW HISTORY

BMJ Open publishes all reviews undertaken for accepted manuscripts. Reviewers are asked to complete a checklist review form and are provided with free text boxes to elaborate on their assessment. These free text comments are reproduced below.

ARTICLE DETAILS

Title (Provisional)

Efficacy of psychotherapy versus pharmacotherapy, or their combination, in chronic depression: study protocol for a systematic review and network meta-analysis using aggregated and individual patient data

Authors

Schramm, Elisabeth; Elsaesser, Moritz; Müller, Julia; Kwarteng, Nana-Adjoa; Evrenoglou, Theodoros; Cuijpers, Pim; Orestis, Efthimiou; Klein, Daniel N; Keller, Martin B.; Furukawa, Toshi A.; Nikolakopoulou, Adriani

---

VERSION 1 - REVIEW

**Reviewer**	**1**
**Name**	**Arena, Andrew**
**Affiliation**	**Black Dog Institute**
**Date**	**04-Nov-2024**
**COI**	**None**

The manuscript is well-written and quite thorough, although certain elements in the method could be further clarified/justified:

Line 152: The introduction states that the definition of 'chronic' varied in the literature between 1-3 years, so there could be clearer justification of why 'at least 2 years' was chosen for the purpose of the review

Lines 186-195: There are no pharmacological search terms included, e.g., around antidepressants, which seems to imply that articles on relevant treatments may not have been retrieved. Given the inclusion criteria includes pharmacological therapies, it's unclear whether these terms were omitted from the search or only the reporting of the search terms – and particularly if the former, why? Also, some psychotherapies of interest don't seem to be captured in the search terms (e.g, 'Metacognitive training'), meaning that articles using only these terms may be omitted from search results. More information is needed before the potential impact of this issue on the study can be known.

Line 247-253: Is any strategy prepared for dealing with articles that report data for more than 1 scale of the same outcome?

Line 295: Is there a statistical basis for excluding outcomes with >50% missing data? Or is this simply a rule implemented for the purpose of consistency within this review? Also noting that 30% missing data is stated in Line 366 for covariate data, and perhaps reasons for why this rule is different for this data could be clarified.

Other notes:

Line 158: Was there any consideration of novel drug/drug-assisted therapies like ketamine or psychedelic-assisted psychotherapy? Or any reason why these were out of scope?

Line 220: Define “AD” upon first use in the main text

**Reviewer**	**2**
**Name**	**Shohaimi, Shamarina**
**Affiliation**	**Universiti Putra Malaysia**
**Date**	**01-Jan-2025**
**COI**	**None**

- In the abstract, consider briefly mentioning the significance of combining individual patient data (IPD) and aggregate data (AD) to highlight the novelty of this approach.

- While the inclusion criteria are well-detailed, it would be helpful to explain why internet-based psychotherapy studies are excluded, as these interventions are becoming increasingly common.

- The use of the Cochrane RoB 2 tool is appropriate. Adding a brief explanation of the process for resolving disagreements between raters would provide more clarity.

- Since the manuscript notes the challenge of small sample sizes for subgroup analyses, it would be useful to include alternative strategies if certain subgroups cannot be analyzed.

- Ensure consistent use of abbreviations (e.g., IPD, AD) throughout the manuscript for readability.

VERSION 1 - AUTHOR RESPONSE

Reviewer 1: Dr. Andrew Arena, Black Dog Institute

***R1:** The manuscript is well-written and quite thorough, although certain elements in the method could be further clarified/justified:*
Thank you for your kind evaluation and your helpful comments.

***R1:** Line 152: The introduction states that the definition of 'chronic' varied in the literature between 1-3 years, so there could be clearer justification of why 'at least 2 years' was chosen for the purpose of the review*
We chose the definition of at least 2 years to align with the DSM-5 criteria for Persistent Depressive Disorder, which consolidates various chronic depression subtypes under a unified diagnostic framework. This approach ensures consistency across included studies and reflects the most widely accepted clinical definitions. It is now clearly stated in the text: "*Depressive courses are defined as chronic when lasting for at least two years (according to the definition of the classification systems of DSM-5 and ICD-11) including persistent depressive disorders…*" (Line 156 ff.)
***R1:** Lines 186-195: There are no pharmacological search terms included, e.g., around antidepressants, which seems to imply that articles on relevant treatments may not have been retrieved. Given the inclusion criteria includes pharmacological therapies, it's unclear whether these terms were omitted from the search or only the reporting of the search terms – and particularly if the former, why?*
Thank you for your comment regarding the inclusion/exclusion criteria for our meta-analysis. Our decision to exclude pharmacotherapy-only studies is grounded in the primary research aim of our study, which is to evaluate the efficacy of psychotherapy in comparison to pharmacotherapy and combination therapy. Including studies that investigate only pharmacotherapy would not directly contribute to this comparative analysis and might introduce heterogeneity that is not aligned with our research objectives. To make this clearer, we changed the title to '*Efficacy of psychotherapy **vs.** pharmacotherapy, or their combination in chronic depression: Study protocol for a systematic review and network meta-analysis using aggregated and individual patient data*' and adapted the abstract and the main text accordingly.
***R1:** Also, some psychotherapies of interest don't seem to be captured in the search terms (e.g, 'Metacognitive training'), meaning that articles using only these terms may be omitted from search results. More information is needed before the potential impact of this issue on the study can be known.*
The omission of certain psychotherapies from the search string is due to their limited impact on the retrieval of additional relevant articles. The search string was developed through a careful iterative process that included testing various search terms (e.g., metacognitive training, acceptance and commitment therapy). Terms that did not yield additional results were excluded again to maintain clarity and avoid redundancy of the search string. One possible explanation for 'metacognitive training' or 'ACT' yielding no additional hits is that these therapies may already be captured by broader terms used in the search, such as psychotherapy or psychological intervention, which may be the case if these search terms were mentioned in the title or abstract. It is also possible that there are limited or no studies explicitly investigating these specific psychotherapies within the scope of the inclusion criteria.
***R1:** Line 247-253: Is any strategy prepared for dealing with articles that report data for more than 1 scale of the same outcome?*
We have updated the manuscript to describe the strategy for articles which report data for more than one scale of the same outcome. In particular, we now state in lines 298 ff: "*In studies where multiple scales are reported, all scales reported in the articles will be extracted and those that can be converted to HAM-D will be used for the analysis. In cases where multiple scales are reported and more than one can be converted to HAM-D, or if neither scale can be converted, the scale that is more commonly used across studies will be selected for consistency and comparability.*"
***R1:** Line 295: Is there a statistical basis for excluding outcomes with >50% missing data? Or is this simply a rule implemented for the purpose of consistency within this review? Also noting that 30% missing data is stated in Line 366 for covariate data, and perhaps reasons for why this rule is different for this data could be clarified.*

The rules are implemented specifically for the purpose of consistency within this review. The 50% cutoff for outcomes was selected based on a previous review on therapy for depression (Karyotaki et al., 2019; https://doi.org/10.1136/bmjopen-2018-026820). In the previous review, however, only covariates that were reported in all studies were included. As we now expect a richer IPD database, this will enable us to apply the same imputation approach for missing covariates. To ensure, though, that the analysis is predominantly supported by observed data rather than the imputations, we selected a lower threshold (30%) for covariate imputation.
R1: *Other notes: Line 158: Was there any consideration of novel drug/drug-assisted therapies like ketamine or psychedelic-assisted psychotherapy? Or any reason why these were out of scope?*
Any eligible studies involving novel therapies that align with our inclusion/exclusion criteria will be integrated into the analysis.
R1: *Line 220: Define "AD" upon first use in the main text*
"AD" is defined as "aggregate data" upon its first mention in the main text (line 139).

Reviewer 2: Dr. Shamarina Shohaimi, Universiti Putra Malaysia

R2: *In the abstract, consider briefly mentioning the significance of combining individual patient data (IPD) and aggregate data (AD) to highlight the novelty of this approach.*
We have updated the abstract to describe the benefits of IPD and AD. In particular, we now state in the abstract: "For studies which do not provide IPD, aggregate data (AD) will be extracted and incorporated in lieu of IPD for the network meta-analysis (NMA), strengthening the evidence base and leveraging all existing evidence regardless of data availability restrictions."
R2: *While the inclusion criteria are well-detailed, it would be helpful to explain why internet-based psychotherapy studies are excluded, as these interventions are becoming increasingly common.*
We excluded exclusively internet-based psychotherapy studies to maintain consistency and homogeneity in the delivery format of psychotherapeutic interventions analyzed in this review. Internet-based psychotherapies, while increasingly common and promising, differ substantially in their structure, intensity, therapist involvement, and patient engagement compared to face-to-face interventions. These differences introduce significant heterogeneity, which could compromise the validity and interpretability of our network meta-analysis results. Furthermore, a recent systematic review in JAMA Psychiatry (Merzhvynska et al., 2023; https://doi.org/10.1001/jamapsychiatry.2023.3861) concludes "*indirect comparisons of FTF and IBT may be problematic because, in terms of reporting prognostic risk factors, samples of RCTs may not be drawn from the same clinical population.*" While we acknowledge the growing relevance of internet-based psychotherapies, their inclusion would require a separate systematic review and meta-analysis to account for their unique features. Future research should explore the efficacy of these interventions specifically, as their accessibility and scalability hold significant potential for addressing treatment gaps in chronic depression.
R2: *The use of the Cochrane RoB 2 tool is appropriate. Adding a brief explanation of the process for resolving disagreements between raters would provide more clarity.*
Thank you for your comment. As stated in the manuscript, disagreements between raters will be resolved through discussion. If consensus cannot be reached, a third reviewer will be consulted. To provide additional clarity, we have now referenced the Cochrane Handbook for Systematic Reviews of Interventions as the methodological framework guiding this process (*Higgins JPT, Thomas J, Chandler J, Cumpston M, Li T, Page MJ, Welch VA (editors). Cochrane Handbook for Systematic Reviews of Interventions version 6.5 (updated August 2024). Cochrane, 2024. Available from www.training.cochrane.org/handbook.*). Furthermore, we have added that all raters involved in the

risk of bias assessment have completed an official Cochrane Workshop on the RoB 2 tool, ensuring a consistent and rigorous approach to evaluating bias across included studies.
R2: *Since the manuscript notes the challenge of small sample sizes for subgroup analyses, it would be useful to include alternative strategies if certain subgroups cannot be analyzed.*
Thank you for highlighting this challenge. We have now added the following at the end of 'Network Meta-Regression (NMR)': "If subgroup analyses cannot be undertaken due to small number of studies, we will conduct separate pairwise meta-analyses for the small subgroups and provide descriptive statistics to offer insight regarding the subgroup. In the particular case where subgroup analyses result in disconnected sub-networks, i.e. there are pairs of treatments that neither direct nor indirect estimates can be derived, we will employ a component NMA (cNMA) in an attempt to connect the disconnected networks consisting of the small subgroup and facilitate analysis." (Line 440 ff.)
R2: *Ensure consistent use of abbreviations (e.g., IPD, AD) throughout the manuscript for readability.*
Abbreviations such as IPD and AD have been standardized throughout the manuscript for improved readability.
